# The investigation of Strongyloides stercoralis seroprevalence in immunosupressed patients in Turkey

**DOI:** 10.3906/sag-1804-16

**Published:** 2019-02-11

**Authors:** Filiz KAYA, Ahmet Çağkan İNKAYA, Ali İhsan ERTENLİ, Osman ABBASOĞLU, Sercan AKSOY, Yakut AKYÖN YILMAZ, Sibel ERGÜVEN

**Affiliations:** 1 Department of Medical Microbiology, Ankara Training and Research Hospital, Ankara Turkey; 2 Department of Infectious Diseases, Faculty of Medicine, Hacettepe University, Ankara Turkey; 3 Department of Internal Diseases Rheumatology Subdivision, Faculty of Medicine,Hacettepe University, Ankara Turkey; 4 Department of General Surgery, Faculty of Medicine, Hacettepe University, Ankara Turkey; 5 Department of Medical Oncology, Faculty of Medicine, Hacettepe University, Ankara Turkey; 6 Department of Medical Microbiology, Faculty of Medicine, Hacettepe University, Ankara Turkey

**Keywords:** *Strongyloides stercoralis*, strongyloidiasis, immunosuppression, serology, parasitic infection

## Abstract

**Background/aim:**

In immunosuppressed patients, strongyloidiasis can be lifethreatening because of hyperinfection or dissemination. Therefore, diagnosis of *S. stercoralis* is important in immunosuppressed patients with chronic strongyloidiasis. In this study, our objective was to investigate the presence of *S. stercoralis* antibodies by an ELISA method in immunosuppressed patients.

**Materials and methods:**

A total of 100 immunosuppressed patients’ sera were included in the study. Forty-two of the patients were receiving immunosuppressive therapies for cancer or being treated for hematopoietic malignancies, 38 of the patients were receiving immunosuppressive drugs for rheumatic diseases, 14 were receiving immunosuppressive therapies for liver transplantation. Two of the patients were being treated for HIV infection and 4 were being treated for hypogammaglobulinemia. As control group, 50 individuals without a known disease were included in the study. The presence of IgG antibodies against *S. stercoralis* was investigated with a commercial ELISA kit.

**Results:**

*S. stercoralis* antibody test was positive in 4 of 100 (4%) sera from immunosuppressed patients. All control patients were negative for *S. stercoralis*.

**Conclusion:**

Strongyloidiasis can be a lifelong chronic infection if not treated. In patients who are going to receive immunosuppressive therapy, it should be tested before treatment, as it can become a disseminated and life-threatening infectious disease.

## 1. Introduction

*S. stercoralis* is a soil-transmitted intestinal nematode that causes strongyloidiasis in humans. It is estimated that 30–100 million people are infected with *S. stercoralis* worldwide. Strongyloidiasis is endemic in tropical and subtropical areas especially where the sanitation conditions are poor (1). 

The infection is acquired through contact with soil that is contaminated with free-living larvae. The larvae proceed via venous circulation, migrate to the lungs, are swallowed and reach the small intestine where they develop into adult parasites. Rhabditiform larvae are excreted in stool and transform into infective filariform larvae. The parasite can cause autoinfection which may lead persistent infections (1,2) . 

Most of the *S. stercoralis *infections are asymptomatic. Symptomatic infection is usually characterized by gastrointestinal symptoms such as diarrhea and abdominal pain or skin manifestations like itching and urticaria (3). 

*S. stercoralis* infection can be severe and life-threatening in patients with immunosuppression. In this case, hyperinfection or dissemination of strongyloidiasis that leads to systemic sepsis, and multiorgan failure may develop. Hyperinfection syndrome is defined as accelerated autoinfection. Disseminated infection occurs when larvae migrate into organs other than the skin, gastrointestinal tract, or lungs. Predisposing risk factors for developing disseminated infection and hyperinfection syndrome are immunosuppressive therapies for immune-mediated disorders, infections with the human immunodeficiency virus (HIV), hematopoietic malignancies, and solid organ transplants (4). 

Diagnosis of asymptomatic *S. stercoralis* infection in immunosuppressed patients is important to prevent life-threatening complications. If strongyloidiasis is diagnosed early, it is easily treatable with oral antihelmintic drugs (4). Because of the low parasite load and irregular larval output, diagnosis may be difficult by stool examination (5). Serological methods such as enzyme-linked immunosorbent assay (ELISA) or indirect immunofluorescent test (IFAT) and molecular tests may be alternative diagnostic methods for rapid detection of *S. stercoralis* infection (6). 

In this study, our objective was to investigate the presence of *S. stercoralis* antibodies in the serum samples by using an ELISA method in immunosuppressed patients. 

## 2. Materials and methods 

### 2.1. Patients 

A total of 100 immunosuppressed patients’ sera were included in the study. In this patient group ranging from 18 to 66 years of age, 52 patients were male and 48 were female. Forty-two of the patients were receiving immunosuppressive therapies for cancers such as lung cancer and breast cancer or being treated for hematopoietic malignancies such as lymphoma. Thirty-eight of the patients were receiving immunosuppressive drugs for rheumatic diseases such as ankylosing spondylitis and rheumatoid arthritis. Fourteen of the patients were receiving immunosuppressive therapies for liver transplantation. Two of the patients were being treated for HIV infection and four of the patients were being treated for hypogammaglobulinemia. As the control group, 50 individuals without a known disease, i.e. apparently healthy, and not receiving immunosuppressed treatment were included in the study. In the control group, there were 29 males and 21 females whose ages ranged from 19 to 63. 

### 2.2. Blood samples

A total of 150 blood samples were collected. A hundred of them were from the patients that are shown in Table and 50 were from the healthy subjects. Sera were separated from blood and stored at −20 °C until analysis. 

### 2.3. ELISA testing 

The presence of IgG antibodies against *S. stercoralis* in sera were investigated by a commercial ELISA kit (DRG Strongyloides IgG, USA) according to the instructions of manufacturers. In brief, serum samples were diluted 1:64 in dilution buffer and added antigen-coated wells. After being incubated at room temperature for 10 min, the wells were washed three times with the diluted wash buffer and 2 drops of enzyme conjugate were added to each well. After incubation at room temperature for 5 min, the wells were washed again and 2 drops of chromogen were added. Following a 5 min of incubation, 2 drops of stop solution were added and the reaction was stopped. The results were read at 450 nm and 0.5 OD units and above were accepted as positive results. Each serum sample was examined twice at two different times. 

All tests were performed in the Parasitology Section of the Department of Medical Microbiology. The study was approved by the Ethical Committee of Hacettepe University.

## 3. Results 

There was no difference between the two groups in terms of age and sex. Epidemiologic and sociodemographic features were similar. 

Out of 100 patients, 24 were diagnosed with ankylosing spondylitis and 14 with rheumatoid arthritis. All of these rheumatology patients were receiving immunosuppressive agents. Four of them were positive for *S. stercoralis* antibodies. Two were diagnosed with ankylosing spondylitis and treated with etanercept, methotrexate, and prednisolone. Two patients had rheumatoid arthritis and were taking infliximab and methotrexate. None of these patients had symptoms, such as diarrhea, abdominal pain, nausea, vomiting, cutaneous lesions, or respiratory symptoms, that point to a parasitic infection. Eosinophil levels were within normal range in all sera-positive patients. There were 42 patients with malignancies in the study. Twenty-four patients were receiving chemotherapy for solid organ tumour and 18 for hematopoietic malignancies. None of them were positive for *S. stercoralis* antibodies. In two AIDS patients with diarrhea, *S. stercoralis* antibodies were negative. Fourteen of the patients were receiving immunosuppressive therapies for liver transplantation. *S. stercoralis* antibodies were not detected in any of them. All control patients were negative for *S. stercoralis *antibody (Table). 

**Table 1:** Clinical conditions and seropositivity of patients.

Clinical condition	Number	S. stercoralis antibody positivity %
Ankylosing spondylitis	24	2
Rheumatoid arthritis	14	2
Solid organ tumour	24	0
Hematopoietic malignancies	18	0
Liver transplantation	14	0
Hypogammaglobulinemia	4	0
HIV-positive	2	0
Total	100	4 %

## 4. Conclusion

*S. stercoralis* infection can be severe and life-threatening in patients with immunosuppression. Predisposing risk factors for developing disseminated infection and hyperinfection syndrome are immunosuppressive therapies for immune-mediated disorders, infections with the human immunodeficiency virus (HIV), hematopoietic malignancies, and solid organ transplants (4). It is reported that serological methods can be used as screening tests in immunosuppressed patients and the sensitivity of ELISA ranges from 80% to 100% (7,8). 

In this preliminary study, our object was to evaluate the *S. stercoralis* serology for the management of probable chronic strongyloidiasis in patients with immunosuppresion. 

Taking high doses of corticosteroids or antiinflammatory agents for rheumatoid diseases is a risk factor for developing severe strongyloidiasis (9). Yanık et al. reported *S. stercoralis* infection in a patient taking corticosteroid and infliximab for several years because of ankylosing spondylitis. The patient had no signs that indicate *S. stercoralis* infection except high levels of IgE and eosinophilia. Histological examination of endoscopic duodenal biopsy revealed *S. stercoralis* larvae (10). There are also hyperinfection syndromes with *S. stercoralis* in rheumatology patients reported in the literature. Yılmaz et al. presented a hyperinfection syndrome in a patient with Behçet’s Disease on immunosuppressive treatment (11). Altintop et al. and Das et al. reported hyperinfection syndromes in patients with rheumatoid arthritis treated with steroids and methotrexate (12,13). In our study, *S. stercoralis* antibodies were found positive in rheumatology patients who were receiving immunosuppressive drugs. None of them had symptoms that point to a parasitic infection. Eosinophil levels were also within the normal range. In these patients, further investigations, such as Baermann’s technique, stool culture, polymerase chain reaction, or endoscopic duodenal biopsy, may be required to detect possible *S. stercoralis* infection. 

In oncology patients, diarrhea is one of the complications of chemotherapy and may be disregarded in terms of parasitic infection. There are reported cases of strongyloidiasis in patients with malignancies receiving immunosuppressive therapies (14). Zueter et al. studied 192 stool and serum samples collected from cancer patients who were receiving chemotherapy. They found that 1 (0.5%) sample was positive for *S. stercoralis* by microscopy, 3 (1.6%) by real-time PCR, 8 (4.2%) by IgG-ELISA. In patients with malignancies, immunosuppressive therapy can be the cause of fatal strongyloidiasis (15). Although we found no seropositivity in oncology patients in our study, it is important to follow up these individuals in terms of developing parasitic infection. 

Strongyloidiasis is reported to be common in AIDS patients in endemic areas. Jongwutiwes et al. reported a case-control study for determining the prevalence and risk factors of *S. stercoralis* infection. They found that strongyloidiasis was seen more often in male patients with eosinophilia and with HIV infection (9). Despite the fact that prevalence rates of infection are higher in the individuals with HIV, some researchers report that hyperinfection syndrome is rarely seen in advanced HIV disease because of increased Th2 response which may protect against severe forms of strongyloidiasis (16-19). In our study, there were two HIV-positive patients and they both had diarrhea. *S. stercoralis* antibodies were found negative in these individuals. 

Several *S. stercoralis* infections in heart, kidney, intestine, liver, and stem cell transplant patients have been reported. In most of the cases, the infection was caused by reactivation of latent infections; in a few cases, it was acquired from the donors. Severe strongyloidiasis in transplant patients reveals high mortality rates (20–24). We did not detect *S. stercoralis* antibodies in patients receiving immunosuppressive therapies for liver transplantation. 

In Turkey, the prevalence of *S. stercoralis* infection is reported between 0% and 4.4% based on stool examination (25). Aksoy Gökmen et al. investigated the incidence of *S. stercoralis* in individuals who live in rural areas and work in the garden; one of 281 volunteers (0.3%) was positive for *S. stercoralis*-IgG antibodies (26). 

To our knowledge, this is the first study conducted to detect the *S. stercoralis* seroprevalance of immunosuppressed patients in Turkey. A total of 150 sera sample were tested for the presence of IgG antibodies against *S. stercoralis.* Four of 100 (4%) sera from immunosuppressed patients were positive. In the control group, all individuals were sera-negative. Limitations of this study are low number of patients and inadequacy of fecal tests due to poor compliance of patients to the repeated stool examinations. Further investigation with molecular techniques may be an alternative diagnostic tool for the detection of *S. stercoralis* in these patients. 

Although there were no signs of parasitic involvement in our sera-positive patients, it might be beneficial to follow up these patients in terms of developing *S. stercoralis* infection. It is important to detect latent *S. stercoralis* infections before administering chemotherapy or before the onset of immunosuppression in patients at risk to prevent severe complications. 

## Acknowledgments

This work was supported by the Research Fund of the Hacettepe University; project number: 1323. Poster presentation of the study was made in the 9th Balkan Congress of Microbiology, 22–24 Oct, 2015. 

## References

[ref0] (2013). Strongyloides stercoralis infection. BMJ.

[ref1] (2011). Strongyloidiasis: a multifaceted disease. Gastroenterol Hepatol (N Y).

[ref2] (2013). Strongyloides stercoralis is a cause of abdominal pain, diarrhea and urticaria in rural Cambodia. BMC Res Notes.

[ref3] (2012). Screening, prevention, and treatment for hyperinfection syndrome and disseminated infections caused by Strongyloides stercoralis. Curr Opin Infect Dis.

[ref4] (2001). Diagnosis of Strongyloides stercoralis infection. Clin Infect Dis.

[ref5] (2013). Serological and molecular detection of Strongyloides stercoralis infection among an Orang Asli community in Malaysia. Parasitol Res.

[ref6] (2016). Diagnosis of Strongyloides stercoralis infection in immunocompromised patients by serological and molecular methods. Rev Inst Med Trop Sao Paulo.

[ref7] (2001). The value of an immunoenzymatic test (enzyme-linked immunosorbent assay) for the diagnosis of strongyloidiasis in patients immunosuppressed by hematologic malignancies. Am J Trop Med Hyg.

[ref8] (2014). Prevalence and risk factors of acquiring Strongyloides stercoralis infection among patients attending a tertiary hospital in Thailand. Pathog Glob Health.

[ref9] (2013). Strongyloides stercoralis in a patient with ankylosing spondylitis: case report (article in Turkish with an abstract in English). Turkiye Parazitol Derg.

[ref10] (2013). Strongyloides stercoralis hyperinfection syndrome in a patient with Behçet’s Disease (article in Turkish with an abstract in English). Turkiye Parazitol Derg.

[ref11] (2010). Strongyloides stercoralis hyperinfection in a patient with rheumatoid arthritis and bronchial asthma: a case report. Ann Clin Microbiol Antimicrob.

[ref12] (2007). Strongyloides hyperinfection in rheumatoid arthritis. Int J Surg Pathol.

[ref13] (2012). Symptomatic chronic strongyloidiasis in children following treatment for solid organ malignancies: case reports and literature review. Trop Biomed.

[ref14] (2014). Detection of Strongyloides stercoralis infection among cancer patients in a major hospital in Kelantan, Malaysia. Singapore Med J.

[ref15] (2010). Strongyloides stercoralis: there but not seen. Curr Opin Infect Dis.

[ref16] (2001). High prevalence of giardiasis and stronglyloidiasis among HIV-infected patients in Bahia, Brazil. Braz J Infect Dis.

[ref17] (2012). Is human immunodeficiency virus infection a risk factor for Strongyloides stercoralis hyperinfection. PLoS Negl Trop Dis.

[ref18] (2004). Why does HIV infection not lead to disseminated strongyloidiasis?. J Infect Dis.

[ref19] (2013). Strongyloides stercoralis and organ transplantation. Case Rep Transplant.

[ref20] (2013). Strongyloides stercoralis transmission by kidney transplantation in two recipients from a common donor. Am J Transplant.

[ref21] (2013). Strongyloides stercoralis infection in an intestinal transplant recipient. Transpl Infect Dis.

[ref22] (2012). Fatal disseminated strongyloidiasis after kidney transplantation. Rev Soc Bras Med Trop.

[ref23] (2013). Fatal strongyloides hyperinfection complicating a gram-negative sepsis after allogeneic stem cell transplantation: a case report and review of the literature. Case Rep Hematol.

[ref24] (2009). An overview of Strongyloides stercoralis and its infections (article in Turkish with an abstract in English). Mikrobiyol Bul.

[ref25] (2016). Investigation of Strongyloides stercoralis seroprevalence in individuals who grow vegetables and gather mushroom in Muğla province in Turkey (article in Turkish with an abstract in English). Turkiye Parazitol Derg.

